# Type 2 Diabetes Induced by Changes in Proteomic Profiling of Zebrafish Chronically Exposed to a Mixture of Organochlorine Pesticides at Low Concentrations

**DOI:** 10.3390/ijerph19094991

**Published:** 2022-04-20

**Authors:** Yan Gao, Hyojin Lee, Sangkyu Lee, Ki-Tae Kim

**Affiliations:** 1 BK21 FOUR Community-Based Intelligent Novel Drug Discovery Education Unit, College of Pharmacy and Research Institute of Pharmaceutical Sciences, Kyungpook National University, Daegu 41566, Korea; gaoyan0723@gmail.com; 2 Department of Environmental Engineering, Seoul National University of Science and Technology, Seoul 01811, Korea; hyojin_lee@seoultech.ac.kr

**Keywords:** organochlorine pesticide mixtures, type 2 diabetes mellitus, zebrafish, proteomics, aldehyde dehydrogenase 2, glyceraldehyde 3-phosphate dehydrogenase

## Abstract

Effect of organochlorine pesticides (OCPs) mixtures on development of type 2 diabetes mellitus (T2DM) and the underlying mechanism, especially at protein levels, are largely unknown. We exposed a mixture of five OCPs to zebrafish at concentrations of 0, 0.05, 0.25, 2.5, and 25 μg/L for 12 weeks. Differentially expressed proteins (DEPs) were quantitatively identified in female zebrafish livers, and its functional study was conducted. The significantly high glucose and low insulin levels were observed only at 0.05 μg/L, linking to the different pattern of DEPs than other concentrations. A total of 1082 proteins was quantified, of which 321 proteins formed 6 clusters in protein dynamics analysis. The enriched pathways in cluster 3 showing distinct pattern of DEPs could explain the nonlinear response at 0.05 μg/L, indicating that OCP mixtures adversely affected proteins associated with mitochondrial function and energy metabolism. We proposed a feasible mechanism that decrease in expression of aldehyde dehydrogenase led to abnormal accumulation of aldehydes, reducing expression of glyceraldehyde 3-phosphate dehydrogenase, and resulting in disruption of glucose homeostasis. Our findings help to better understand the causality of T2DM by exposure to OCP mixtures and to identify biomarkers in the protein expression level.

## 1. Introduction

In spite of banned production and use, persistent organic pollutants (POPs) have been still present in the natural and life environment, causing an intentional or unintentional exposure to the flora and fauna. Human is also at risk of POPs that are exposed via food consumption or environmental pollution and then stored for a long time in the lipid-rich tissues [1,2,3]. This accumulated POPs in adipose tissue can exert diverse toxicological effects such as carcinogenesis, endocrine disruption, neurobehavioral impairment, and particularly metabolic dysfunction [4,5,6].

Type 2 diabetes mellitus (T2DM) is a metabolic disease characterized by pancreatic β-cell dysfunction and insulin resistance. It was reported that chronic exposure to POPs can contribute to the onset and development of T2DM [7]. Among a variety of POPs, organochlorine pesticides (OCPs) are known to have a high positive association with T2DM prevalence or impaired insulin actions in human studies [8,9]. In experimental studies as well, the evidences for contribution of OCPs to T2DM were found. The short-term gestational exposure to dichlorodiphenyltrichloroethane (DDT) caused glucose intolerance and hyperinsulinemia in mice adult offsprings [10]. The acute exposure to dichlorodiphenyltrichloroethylene (DDE), a metabolite of DDT, induced hyperglycemia in C57BL/6H mice [11], and other OCP substances, e.g., endosulfan, lindane, and aldrin, disrupted hepatic metabolites in the glucose metabolism such as citric acid cycle in the HepG2 cells [12]. Despite findings on the diabetic induction by OCPs, the underlying mechanisms in particular at protein level remain still unknown. Quantitative proteomic analysis helps in identifying biomarkers and understanding mechanisms of toxicity and disease, such as dysfunctional lipid metabolism, pancreatic impairment, and neurological disorders [13,14,15]. A systematic proteomics investigation therefore is necessary to explore mechanism at protein levels that underlies the incidence of T2DM caused by OCP exposure.

Zebrafish have been positioned as model organism to explore the mechanism of disease including metabolic disorders like obesity and T2D. While most previous studies have utilized zebrafish to discover genes and lifestyle indicative of disease and to visualize relevant tissue or gene [16], a recent study using zebrafish showed that exposure to chemicals can be a risk factor of metabolic disease [17]. To support epidemiological consequences, incidence of disease by chemical exposure is needed to be investigated with chronic exposure and a low concentration. With regard to realistic exposure scenarios, various OCPs accumulated in adipose tissue behave as a mixture, and they are continuously released into the body at low concentration levels [18]. To date, zebrafish are efficient for resource-efficient chronic testing and obtained evidences can be translated into conserved biology in human because of well-annotated genome and high genetic and physiological homology with human [19].

It was confirmed from our prior study that OCPs mixtures nonlinearly cause an increase of blood glucose and a deficiency of insulin at low concentration in female zebrafish [20]. Further exploration is needed to understand a reason why OCPs mixtures showed a nonlinear response to T2DM. Also, the search for remarkable biomarkers is needed to develop surrogate endpoints and efficiently evaluate T2DM caused by chemical exposure. Application of the proteomics approach helps in demystifying molecular mechanisms as well as discovering biomarkers for diagnosis, prognosis, and treatment of T2DM [21]. Therefore, the aim of this study was to investigate proteome profiling of zebrafish livers using isobaric tags multiplexed quantitative proteomics. We exposed the mixtures of five predominant OCPs including p,p’-DDT, heptachlor, β-hexachlorocyclohexane (β-HCH), hexachlorobenzene (HCB) and chlordane [22,23,24] to zebrafish adults for 12 weeks. The differentially expressed proteins (DEPs) were identified and their relative amounts were quantified in OCP mixtures-exposed groups using LTQ Orbitrap Velos mass spectrometer (MS). Global quantitative proteomic analysis coupled with bioinformatics tools on DEPs was performed to assess the changes in protein dynamics and function. In addition, key proteins, among DEPs identified after exposure to OCP mixtures, were chosen to propose the decisive pathway to address phenotypic traits of T2DM and nonlinear response of OCPs mixture at protein level in zebrafish.

## 2. Materials and Methods

### 2.1. Chemicals

Five OCPs substances, including chlordane (CAS No. 12789-03-6, 409.78 molecular weight (MW)), heptachlor (CAS No. 76-44-8, 373.32 MW), p,p’-DDT (CAS No. 50-29-3, 353.49 MW), β-HCH (CAS No. 319-85-7, 290.83 MW), and HCB (CAS No. 118-74-1, 284.78 MW), were purchased from Sigma Aldrich (St. Louis, MO, USA). Dimethyl sulfoxide was purchased from Sigma Aldrich (St. Louis, MO, USA) and as a solvent 0.1% of itself was included in all groups including a control group. The five OCPs were mixed with 1:1:1:1:1 equal ratio on μg/L, their detected concentrations were addressed in prior study [20]. A ratio of 1:1:1:1:1 was applied based on the results that inhibition of glucose metabolism was similar in 5 OCPs [25,26].

### 2.2. Zebrafish Husbandry

Zebrafish adults (*Danio rerio*, AB-wild type) were in-house cultured in a continuously flow-through system (Tecniplast, Buguggiate, Italy) with 28 ± 0.5 °C and a cycle of 14 h light and 10 h dark in the laboratory at Seoul National University of Science & Technology. Commercial dried food (Gemma Micro, Skretting, Fukuoka, Japan) was supplied to zebrafish after weighing constant amount of 5 mg per fish three times a day for six days a week. Healthy zebrafish aged 10 to 13 months without any injury in the gill, pectoral or dorsal fins were used in the experiments.

### 2.3. Chemical Exposure

The exposure scheme and conditions were detailed in [20]. Briefly, a total of 540 male and female zebrafish were randomly divided into 5 groups, and 90 fish for each concentration and 30 fish per replicates with 1:1 male/female ratio were exposed to OCP mixtures at concentrations of 0, 0.05, 0.25, 2.5, and 25 μg/L, that below reference values of each OCP, under chronic exposure conditions (i.e., 12 weeks) [27,28]. The concentration of 25 μg/L (equated to 75 nM) was made by mixing 5 μg/L of each five OCPs. The chronic exposure to OCP mixtures was conducted in an automatically flow-through exposure system (TOX-MIX II, HANALAB, Daejeon, Korea) in which exposure concentrations were maintained by mixing the stock solution (1000 times higher than the desired concentration) for each concentration and culture media. The flux of water was optimized for circulating 6 L volume of each tank three times a day. The exposure experiment was performed at Korea Testing and Research Institute approved as Good Laboratory Practice with the guidelines for animal welfare and ethics. Although male and female zebrafish were exposed together, as a further experiment of a previous study [20], males showing no significant phenotypes were excluded from the experimental subject in this study. Once the exposure was completed, all zebrafish was fasted over 8 h and anesthetized with the chilly ice (<0 °C). The blood collection and liver dissection were performed quickly, and the weight of individual livers was measured right after separation. All samples were kept at −80 °C until analysis. 

### 2.4. Fasting Glucose and Insulin Measurement

Approximatively 8~10 μL of blood was collected from individual fishes, and 2 μL was used to measure fasting glucose level using OneTouch blood glucose meter (OneTouch^®^ Ultra^®^, LifeScan, Inc., Johnson & Johnson, Irvine, CA, USA). The residual blood was pooled for each replicate and thus three blood samples were prepared in each concentration. All samples were centrifuged under 13,200× *g* with 4 °C and separated into plasma. The plasma insulin level (ng/mL) was measured in triplicates per a concentration with Fish insulin ELISA kit (Cusabio Biotech Co. Ltd., New York, NY, USA).

### 2.5. Protein Extraction from Zebrafish Liver Tissue

The whole proteins were extracted from female zebrafish livers (100~280 μg) pooled from each concentration. To remove the residual blood cells, pooled zebrafish livers were rinsed twice with ice-cold phosphate buffered saline (PBS, pH 7.4). Washed livers were transferred into RIPA lysis buffer (Thermo Fisher scientific, Waltham, MA, USA) containing 1% (*v*/*v*) of protease inhibitor (Thermo Fisher scientific, Waltham, MA, USA) and 1% (*v*/*v*) phosphatase inhibitor (Thermo Fisher scientific, Waltham, MA, USA), respectively. Liver tissues were first homogenized using a mini hand-held homogenizer (Hangzhou Miu Instruments Co., Ltd., Zhejiang, China), and then sonicated for 5 circles (10 s/circle) using ultrasonic instrument (Sonics & Materials, Inc., Newtown, CT, USA). Homogenates were kept at 4 °C thermostatic chamber for 40 min and then centrifuged under 12,000× *g* with 4 °C for 10 min. Bicinchoninic acid assay (Thermo Fisher Scientific, Waltham, MA, USA) was used for protein concentration quantification. Lysates were stored at −80 °C freezer until analysis.

### 2.6. Biological Tryptic Digestion

After protein quantification, 200 μg of proteins from each treatment was taken for in-solution digestion. Peptides were prepared via digestion, purification and trypsin digestion as follows. Protein lysate was reduced in 5 mM dithiothreitol (DTT) for 55 °C for 45 min, alkylated in 15 mM in iodoacetamide (IAA) at room temperature for 30 min in the dark. DTT and IAA were prepared in 25 mM ammonium bicarbonate (ABC) buffer. Protein was predicated with 10% (*v*/*v*) trichloroacetic (TCA) acid in 4 °C for overnight. Protein mixture was centrifuged at 4 °C at 12,000× *g* for 10 min, and the supernatant was abandoned. To remove the residual acid in the protein, pellets were washed in −20 °C acetone twice. Washed pellet was air-dried and re-dissolved in 50 mM tetraethylammonium bromide (TEAB). Trypsin (Promega, Madison, WI, USA) was added to the protein samples for trypsin digestion as a ratio of 1:50 (*w*/*w*), digestion was reacted at 37 °C overnight. Peptide concentrations was measured by the quantitative colorimetric peptide assay (Thermo Fisher Scientific, Waltham, MA, USA) following user’s manual.

### 2.7. TMT Labeling and Peptide Fractionation

TMT Labeling procedure was followed to our previous study [29]. The amount of 40 μg of peptides from each treatment was taken to react with Tandem Mass Tag (TMT) 6-plex reagents (Thermo Fisher Scientific, Waltham, MA, USA). Prior to labeling, TMT reagents were equilibrated to room temperature (RT) for 10 min. Then, 41 µL of acetonitrile (ACN) was added to the TMT reagents, 20.5 μL of TMT reagent was added to 40 μg proteins and incubated for 1 h. Labeling reaction was quenched by adding 4 μL of hydroxylamine (5%) in the RT for 15 min. After quenching labeling reaction, differently labeled peptide were pooled together and dried in Speed-vacuum. To increase the sequence coverage, peptides were fractionated using High pH Reversed-Phase Peptide Fractionation kit (Thermo Fisher Scientific, Waltham, MA, USA). Elution buffers were consisted of 0.1% trimethylamine and 5, 10, 12.5, 15, 17.5, 20, 22.5 25 and 50% acetonitrile (ACN), respectively. Prior to use, the column was conditioned by acetonitrile and trifluoroacetic acid (TFA, 0.1%). Then, the soluble peptides were loaded on the column, and liquid chromatography (LC)-MS grade water was utilized to wash away any residual salts. An additional wash step was performed for removal of the unreacted TMT reagent using 5% ACN in 0.1% TFA. After completely washing the column, peptides were sequentially eluted and dried in Speed-vacuum. Peptides were desalted using C18 Zip-tip (Sigma-Aldrich, St. Louis, MO, USA) prior to LC-MS/MS analysis.

### 2.8. Comparative Analysis of Proteins Using LC-MS/MS

A total amount of 500 ng desalted peptides were dissolved in 2.5 μL solvent A (2% ACN and 0.1% formic acid) and injected to LC-MS. For LC analysis, peptides were loaded on a house packed C12 column (Phenomenex, Torrance, CA, USA). Peptide were separated by a 75 min gradient: start at 5% solvent B (100% ACN with 0.1% formic acid), from 5% to 8% in 2 min, from 8% to 28% in 41 min, from 28% to 90% in 8 min, and 90% in 15 min for maintenance, at a flow rate of 300 nL/min. For MS based proteomic analysis, LTQ Orbitrap Velos (Thermo Fisher Scientific, Waltham, MA, USA) was operated in a positive ion mode. MS data were collected from data-dependent (DDA) mode in the mass range 300–1800 *m*/*z*, resolution at 60,000, maximum IT was 100 ms, electrospray voltage at 2.0 kV, automatic gain control (AGC) target value of 1.0 × 10^6^. TMT labeled peptide was fragmentated in higher-energy collisional dissociation (HCD) mode: 7500 resolution, 40% normalized collision energy, AGC target value of 1.0 × 10^6^. The DDA produced top 10 ions from full scans were performed MS^2^, the maximum IT was 60 ms for MS^2^. Technical duplicated experiments were conducted. The generated MS/MS spectra were searched in MaxQuant (Version 1.5, Max Planck Institute of Biochemistry, Am Klopferspitz, Planegg, Germany) with a peptide FDR < 1%. Pearson correlation between technically replicated experiments was visualized as scatterplot.

### 2.9. Immunoblotting

Zebrafish protein lysate (10 μg) was treated with 4× sodium dodecyl sulfate (SDS) buffer at 95 °C for 5 min. Denatured protein can be separated by 8% SDS-polyacrylamide gel electrophoresis (PAGE) at a voltage of 120 V. Proteins were electro transferred to a PVDF membrane (Roche, Basel, Switzerland) for 2 h at a voltage of 90 V on the ice. Transferred membranes were blocked using 5% BSA in Tris-buffered Saline, 0.1% Tween^®^ 20 Detergent (TBST) (137 mM sodium chloride and 20 mM Tris) for 2 h at RT with shaking. Transferred membranes were incubated with antibodies of anti- aldehyde dehydronenase 2 (ALDH2) (Abcam, Cambridge, UK), anti- glyceraldehyde 3-phosphate dehydrogenase (GAPDH) (Cusabio Biotech, Wuhan, China), and anti-β-actin (Cell Signaling Technology, Danvers, MA, USA) at 4 °C overnight. Membranes were washed with TBST three times with gentle shaking, 10 min intervals were given for each washing step. Membranes were incubated in horseradish peroxidase -linked secondary antibody (Cell Signaling Technology, Danvers, MA, USA) for 2 h at RT, accompanied with another gentle shaking. Membranes were again washed with TBST in the same manner abovementioned. For membrane visualization, membranes were wetted by 1 mL ECL reagent (GE Healthcare, Chicago, IL, USA). Western blot images were captured using an Image Quant LAS 4000 mini (GE Healthcare, Chicago, IL, USA).

### 2.10. Peak Alignment and Data Search

Generated MS2 spectrums were searched by MaxQuant engine (Version.1.5). Zebrafish (*Danio rerio*) database was downloaded from UniProt (https://www.uniprot.org/proteomes/UP000000437, accessed on 14 April 2022) in a fasta format, including the reverse decoy database. Acetyl (protein N-term) and oxidation (M) were added for variable modification. The peptide FDR was set at 0.01. In the produced data files, the protein having score <40 and razor+ unique peptides <2, were filtered. Proteins only identified by site and the reverse database, and potential contaminants were removed from the proteins listed and grouped in this study. Protein quantification was estimated by an intensity-based algorithm to define the up-regulation or down-regulation. Here we defined that the ratio >1.5 is up-regulated, on the contrary, the ratio <0.666 is down-regulated.

### 2.11. Conservation Analysis

In order for identifying the homologous protein between *Danio rerio* with Homo sapiens, protein BLAST (BLASTp) was utilized to analyze a protein-protein BLAST [30]. In the blast program, a non-redundant protein sequence database of UniProt Knowledgebase/Swiss-Prot (UniProtKB/Swiss-Prot) was chosen and matched with Homo sapiens database downloaded from Uniprot (https://www.uniprot.org/proteomes/UP000005640, accessed on 14 April 2022) as a fasta format. A max target sequence was set as 100, and expected threshold was 1 × 10^−6^.

### 2.12. Bioinformatic Analysis

Functional annotations were carried out using Gene ontology (GO) terms database and Kyoto Encyclopedia of the Gene and Genomes (KEGG) pathways. GO enrichment analysis was used to facilitate the biological interpretation of the identified proteins. GO annotation dataset was derived from DAVID (https://david.ncifcrf.gov/, accessed on 14 April 2022), including three ontologies: cellular component (CC), biological process (BP), GO molecular function (MF) of a gene set product. Highly enriched or depleted GO term was determined by Fisher’s exact test *p* value in a two-tailed test. To investigate the dynamic change of proteins, a fuzzy c-means (FCM) algorithm was performed [31]. FCM algorithm is a statistic method for bioinformatics, clustering the omics data based on the similarity of expression patterns and minimizing the variation within each cluster. That is, FCM algorithm was efficiently used for grouping proteins expressed in a similar pattern into the same cluster. 

## 3. Results and Discussion

### 3.1. Fasting Glucose and Insulin Level Analysis

**Figure 1:**
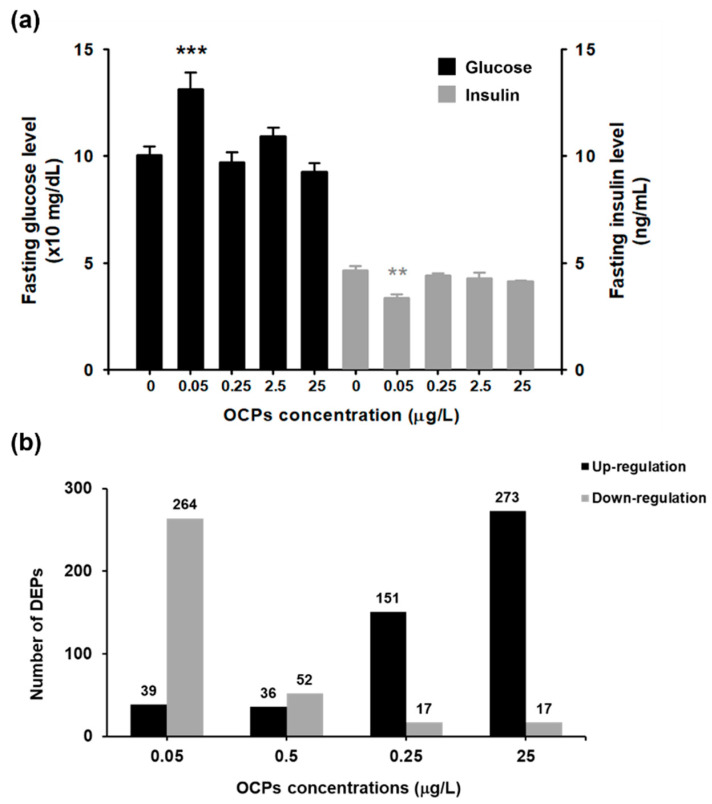
Blood analysis and protein identification in zebrafish exposed to organochlorine pesticide mixtures at concentrations of 0.05, 0.25, 2.5 and 25 μg/L: (**a**) The fasting glucose level (×10 mg/dL, black) and fasting insulin level (ng/mL, gray) were described with the color difference and designed by *** *p* < 0.001 and ** *p* < 0.01; (**b**) The number of up- (black) and down-regulated (gray) proteins was shown.

The fasting glucose and insulin levels were measured to assess the abnormal blood glucose regulation. We found in female that fasting glucose level was significantly increased only at 0.05 μg/L OCP mixtures, at which the insulin was significantly reduced (Figure 1a). Following analysis of protein profiling was conducted with female zebrafish because no significant changes in fasting glucose and insulin levels were observed in male (data not shown in this study). We speculate that the differences in the xenobiotic detoxifying metabolic profiles between male and female may contribute to this sexual dimorphism [32]. Likewise, it was previously reported that the association between exposure to OCPs and T2DM was significant in women than men [33]. Hyperglycemia and insulin deficiency are hallmarks of T2DM; thus, it was concluded that exposure to OCP mixtures induced the phenotypic traits of T2DM in female zebrafish. More importantly, this phenotype was observed at lower concentrations, in a nonmonotonic manner, than what have been tested in previous studies. Having not ever found in experimental studies using animal models, these findings correspond well with human studies reporting the nonlinear association between OCPs concentrations and T2DM in the general population [34]. We hypothesize that this nonlinear inducement of T2DM-related effect is due to hormetic response under stress conditions of chemical exposure [35], often observed by endocrine disrupting chemicals. The activated self-adaptation in response to a high concentration of harmful chemicals restores biological function, inversely a low concentration, in particular with chronic exposure, can be attributable to more adverse effects [36]. 

### 3.2. TMT-Based Proteomic Quantification in Zebrafish Liver

To investigate the DEPs of zebrafish exposed to OCP mixtures, protein dynamics were quantified using TMT-based comparative proteomics. Prior to proteomic analysis, the protein pattern was first examined by SDS-PAGE (Figure S1). The equivalent protein was loaded on SDS-PAGE, showing that the resolved patterns were similar among samples except 0.05 μg/L in which the band was slight weak at the molecule weight of 30 and 100 kDa. In addition, the generated MS/MS spectra were analyzed to confirm the consistency of technical duplication using the MaxQuant search engine (Version 1.5) with a peptide FDR < 1%. The person correlation of technically replicated experiments exhibited R values from 0.72 to 0.85, which indicates a good reproducibility. In the analysis of individual protein dynamics, a total of 1420 unique proteins was identified and 1082 unique proteins were quantified as listed in Supplemental Dataset 1 (XLSX). The protein dynamics were estimated by an intensity-based algorithm that calculates the relative intensity of a reporter ion in groups treated with OCP mixtures compared to the control group. The number of DEPs among concentrations was shown in Figure 1b. The number of up-regulated proteins was increased along with the concentrations, whereas the number of down-regulated proteins exhibited an opposite tendency, the greatest number of down-regulated proteins was displayed at 0.05 μg/L.

These evidences indicate that a low concentration of OCP mixtures influenced on DEPs with a different mechanism compared to their high concentrations, linking to a nonlinear phenotypic response in glucose and insulin levels. We conducted GO enrichment and KEGG pathway analysis for all DEPs quantitatively identified (Figure S2). The pathways in carbon metabolism, biosynthesis of antibiotics, and ribosome were stimulated whereas fatty acid degradation, peroxisome proliferator-activated receptor signaling, and fatty acid metabolism were inhibited. By using DEPs only at 0.05 μg/L, we additionally conducted GO enrichment (Figure S3). For up-regulated proteins, lysosome and tight junction pathways were functionally annotated, and for down-regulated proteins, pathways in metabolic pathways, ribosome, and degradation of aromatic compounds were annotated. The functional network of up- and down-regulated DEPs at 0.05 μg/L was described in Figure S4.

### 3.3. Evolutionary Conservation Protein between Zebrafish and Human

To identify the homologous proteins between zebrafish and homo sapiens, BLASTp analysis was performed with a total of 23 proteins consistently up- or down-regulated at all concentrations. As a result, we identified 22 conserved proteins in human (Table S1). Of the identified proteins, pentaxin was up-regulated at all concentration of OCP mixtures. Pentaxin is the homologue of C-reactive protein (CRP) in human, which was reported to be up-regulated in diabetes patients [37]. CRP is synthesized in the liver with reaction to factors released from macrophages and adipocytes [38], then sensitively responds to the most forms of inflammation [39]. Although the causality of CRP and diabetes remains unclear, we speculate that the elevated expression of CRP could trigger the immune reaction in zebrafish following exposure to OCP mixtures. The chronic inflammation-induced endothelial dysfunction and insulin resistance were known to cause the incidence of metabolic disorders such as obesity and T2DM [37,40]. Secondly, cytochrome P450, family 2, subfamily X, polypeptide 10.2 (CYP2x10.2) and diazepam-binding inhibitor were down-regulated at all concentrations. The protein CYP2x10.2 is homologous to CYP2J2 in human, which was reported to attenuate metabolic dysfunction related to insulin resistance in diabetic mice [40]. It implies that the down-regulation of CYP2x10.2 exerted the negative effect on the glucose metabolism in zebrafish upon exposure to OCP mixtures.

### 3.4. Dynamic Protein Cluster Analysis

**Figure 2:**
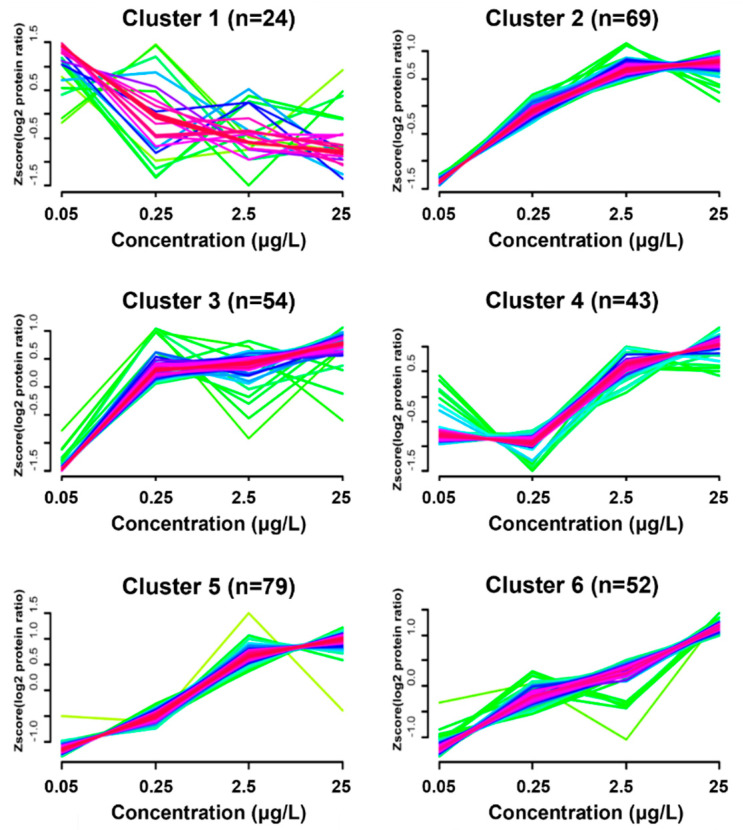
Dynamics clustering analysis of proteins quantified at 0.05 μg/L organochlorine pesticide mixtures. (n = number of proteins included in each cluster).

Based on protein dynamics analysis that identified and quantified 1082 proteins, we applied the FCM algorithm for partitioning of them into different clusters. The comparative analysis of changes in protein expressions resulted in the formation of six distinct clusters (Figure 2). Among 517 DEPs, a total of 321 quantified proteins was included in the clusters, and the number of proteins contained in the cluster 1~6 was 24, 69, 54, 43, 79, and 52, respectively. Hierarchical clustering analysis on the fluctuation in protein expressions among concentrations revealed the specific pattern. Proteins in cluster 1–6 were provided in Supplemental Dataset 2 (XLSX). We observed that the dynamic pattern of protein expression in clusters of 2, 4, 5 and 6 increased in proportion to concentrations, while the pattern in cluster 1 was unordered. Interestingly, in cluster 3, the protein expression was outstandingly lower at 0.05 μg/L than other concentrations, it was increased and leveled off with increasing concentrations This fact is matched with the numerical distribution of down regulated proteins in Figure 1b, showing a large number of down-regulated proteins in 0.05 μg/L group. Accordingly, we consider that the proteins included in the cluster 3 is informative for understanding OCP mixtures caused hyperglycemia.

### 3.5. Biological Functional Analysis

**Figure 3:**
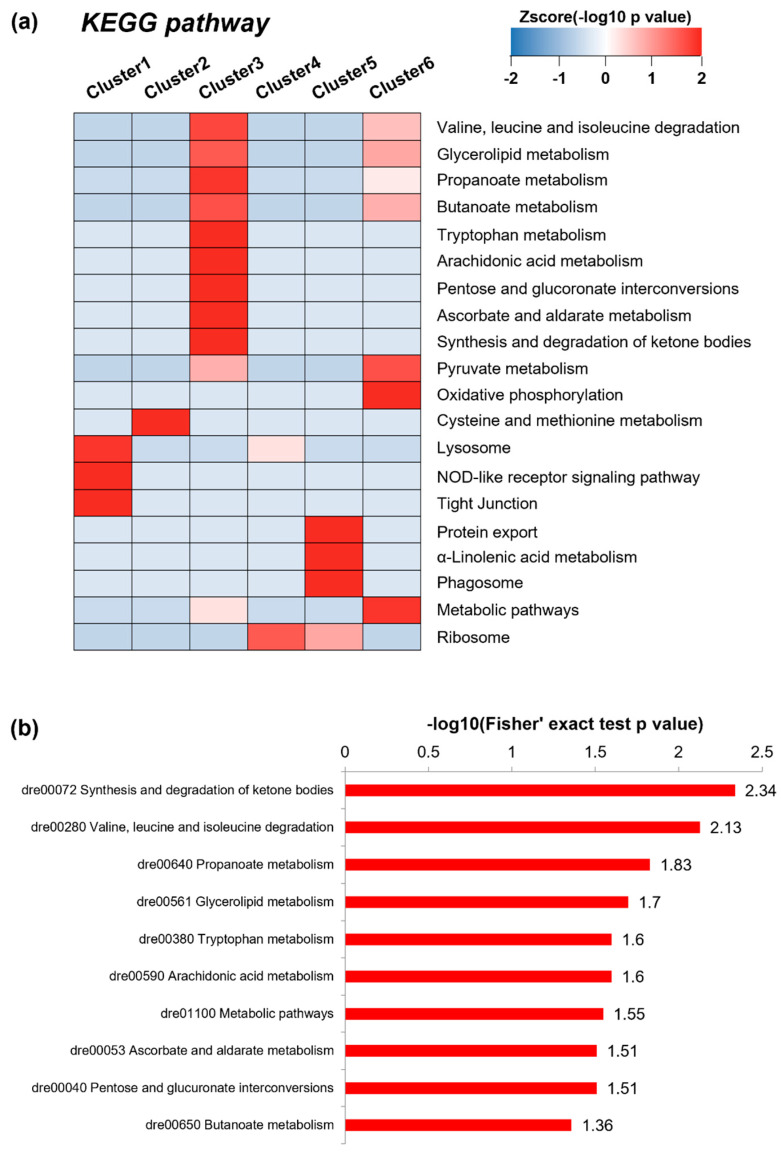
(**a**) KEGG pathway-based enrichment analysis in clusters 1~6. The enrichment efficiency was evaluated by −log 10 of *p* values. (**b**) KEGG pathway-based enrichment analysis using proteins grouped in cluster 3. The Fisher’s exact test *p* value was used to evaluate the enrichment efficiency.

KEGG-based enrichment analysis was conducted to compare overall enriched pathways in clusters 1~6. It was observed that the listed pathways in cluster 3 were distinct from other clusters (Figure 3a). We further performed KEGG-based enrichment analysis for differentially clustered proteins only in cluster 3 (Figure 3b). Some pathways were enriched in an opposite way among clusters. In Figure 3a, the ribosome pathway was lowly enriched in clusters 1, 2, 3 and 6; conversely it was highly enriched in clusters 4 and 5. Likewise, the ribosome pathway was highly enriched when DEPs at all concentrations were considered (Figure S2); however, it was lowly enriched when DEPs in only cluster 3 were considered (Figure S3). It means that the ribosome-related pathway may be less relevant in inducing hyperglycemia. This phenomenon could be one of grounds for our approach that cluster 3 discerned by proteins dynamics was selectively analyzed is favorable.

We found that the enriched proteins in cluster 3 were mainly involved in pathways in synthesis and degradation of ketone bodies, energy metabolism, and metabolism of amino acids such as valine, leucine, and isoleucine. The ketogenesis is the major process to synthesize ketone bodies which are generated by a series of process. Fatty acids are converted to acetyl-CoA with fatty acid β-oxidation and then acetyl-CoA to ketone bodies in the mitochondrial matrix of liver cells [41]. It is well-known that mitochondria produce ATP via oxidation-reduction system, such as fatty acid oxidation and oxidative phosphorylation. Also, above mentioned amino acids play an essential role in glucose uptake and ATP synthesis [42], and they were reported to interact with lipid metabolism and glucose homeostasis in developing T2DM [43]. These evidences lead us to reach the conclusion that proteins enriched in cluster 3 are closely associated with mitochondrial function and energy metabolism. As shown in Figure S3, the enriched pathways, analyzed with DEPs at 0.05 μg/L, from down-regulated proteins, were associated with dysregulation in mitochondrial function and energy metabolism. Indeed, we observed in the same experiment that exposure to OCP mixtures inhibits mitochondrial complex III and IV activities and reduces mitochondrial protein amount in zebrafish livers, both of which are the signature of mitochondrial dysfunction [20]. In addition, it was confirmed by transcriptomics analysis in our prior study that the genes encoding proteins involved in the cluster 3 were significantly changed at 0.05 μg/L. These genes were associated with valine, leucine and isoleucine degradation, pentose and glucuronate interconversions, ascorbate and aldarate metabolism, arachidonic acid metabolism, metabolic pathways, propanoate metabolism, and glutathione metabolism [20].

In the enrichment analysis based on both GO and KEGG database, the protein ALDH2 attracted our attention. ALDH2 was significantly down-regulated at 0.05 μg/L. ALDH2 was reported to be associated with the diabetes complications [44], and its essential role in oxidative pathway of alcohol metabolism involved in acetaldehyde detoxification was discovered [45]. A meta-analysis confirmed that 208 of 515 patients exhibited the association between inactivity of ALDH2 and maternal inheritance of diabetes [46]. In the clinical features of diabetes, this maternal inheritance of diabetes is common in the group inactivating ALDH2 [47], and ALDH2 expression is decreased in the pulmonary of induced diabetic rat [48]. As the deficiency of ALDH2 in mitochondria causes oxidative stress [49], the inhibition of ALDH2 could be linked to mitochondrial dysfunction. Although the causality between inactivation of ALDH2 and diabetes is not fully understood, these evidences support that the inactivity of ALDH2 contributed to the hyperglycemia induced via impaired mitochondrial function and energy metabolism by exposure to OCP mixtures. More mechanistic study is necessary to unravel the molecular pathway that underlies inhibition of ALDH2 after exposure to OCP mixtures. 

In addition, we focused on GAPDH protein. It was significantly down-regulated only at 0.05 μg/L OCP mixtures. Previous studies have reported the association between GAPDH activity and diabetes. For example, the diminished GAPDH activity is observed in embryos of the maternally induced diabetic rat [49] as well as in diabetes rats [50]. As an important enzyme for energy metabolism, GAPDH participates in the sixth step of the glycolysis. In response to excessive reactive oxygen species-induced oxidative stress, GAPDH could be suppressed by acrolein41 and nitric oxide [51]. The accumulation of lipid peroxidation generated by 4-hydroxynonenal could inhibit the expression of GAPDH [20,52]. 

**Figure 4:**
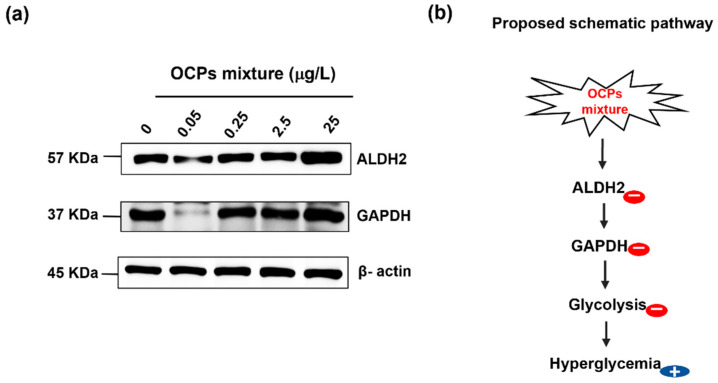
Validation of aldehyde dehydrogenase (ALDH2) and glyceraldehyde 3-phosphate dehydrogenase (GAPDH) expression and proposed mechanism of OCPs-induced hyperglycemia in zebrafish: (**a**) Western blot analyses of ALDH2 and GAPDH expression at different concentrations of organochlorine pesticide mixtures; (**b**) A proposed pathway underlying hyperglycemia -related effects induced in zebrafish.

Western blot analysis was performed to validate the decrease in both ALDH2 and GAPDH expression levels at tested concentrations (Figure 4a). Our results revealed that the lysate of zebrafish liver contained the lowest expression of ALDH2 as well as GAPDH at 0.05 μg/L. The reduced expression of ALDH2 was also appeared at 0.25 and 2.5 μg/L, but these concentrations showed no obvious difference in GAPDH expression compared to the control. The significant reduction in GAPDH expression only at 0.05 μg/L is consistent with other findings in this study. Collectively, from biological functional analysis, we proposed the possible cause of hyperglycemia induced by exposure to a low concentration of OCP mixtures in zebrafish. As illustrated in Figure 4b, the inhibited ALDH2 could induce abnormal accumulation of aldehydes and lead to GADPH reduction, ultimately resulting in a disruption of glucose homeostasis.

## 4. Conclusions

This study is the first study to analyze the perturbation of proteome dynamics and suggest proteomics-based biomarkers, using an adult zebrafish model that exhibits T2DM-related effects caused by chemical mixtures. This study reveals that the alterations of proteomic profiles can be caused by chronic exposure to a low concentration of OCP mixtures in a nonmonotonic manner, which supports the nonlinear associations between POPs mixtures and T2DM as reported in human studies. We demonstrate mitochondrial dysfunction and energy homeostasis impairment are determinant pathways in developing T2DM at the protein level and propose a feasible mechanism for hyperglycemia. More research is followed to discover novel protein biomarkers and related pathways in the pathogenesis of T2DM caused by chemical exposure.

## Data Availability

The datasets used and/or analyzed in the current study are available from Supplemental Materials.

## Supplementary Materials

The following supporting information can be downloaded at: https://www.mdpi.com/article/10.3390/ijerph19094991/s1, Table S1: Protein homology between zebrafish and human, Figure S1: Verification of protein pattern duplication efficiency, Figure S2: GO enrichment analysis and KEGG- and Interpro-based enrichment analysis for proteins consistently up- and down-regulated at all tested concentrations, Figure S3: GO enrichment analysis of proteins in cluster 3, Figure S4: The functional protein association networks of DEPs up- and down-regulated at 0.05 μg/L. The list of all identified and quantified proteins (Supplemental Dataset 1) (XLSX) and list of proteins in each cluster (Supplemental Dataset 2) (XLSX) are provided additionally.
