# Supplementary material for: Type 2 Diabetes Induced by Changes in Proteomic Profiling of Zebrafish Chronically Exposed to a Mixture of Organochlorine Pesticides at Low Concentrations
Source: Int J Environ Res Public Health. 2022 Apr 20;19(9):4991. doi: 10.3390/ijerph19094991 (PMC9100612; doi:10.3390/ijerph19094991)

Supplemental Materials

Type 2 diabetes induced by changes in proteomic profiling of zebrafish chronically exposed to a mixture of organochlorine pesticides at low concentrations

Yan Gao[1,a], Hyojin Lee[2,a], Sangkyu Lee[2,#], Ki-Tae Kim[1,*]

[1]BK21 FOUR Community-Based Intelligent Novel Drug Discovery Education Unit, College of Pharmacy and Research Institute of Pharmaceutical Sciences, Kyungpook National University, Daegu 41566, Republic of Korea

[2]Department of Environmental Engineering, Seoul National University of Science and Technology, Seoul 01811, Republic of Korea

[a]These authors contributed equally to this work.

[#]Co-Corresponding Author

***Corresponding Author:**

Ki-Tae Kim, Ph. D.

Associate Professor, Department of Environmental Engineering

Seoul National University of Science and Technology

Phone: +82-2-970-6642

Fax: +82-2-970-5776

Table S1. Protein homology between zebrafish and human.

Zebrafish		Ratio					Human	
Uniport no.	Description	0.05/VH	0.25/VH	2.5/VH	25/VH	Regulation	Uniport no.	Description
E7F103	Cytochrome P450, family 2, subfamily X, polypeptide 10.2	0.1408	0.3243	0.3546	0.4793	down	P51589	Cytochrome P450 2J2
E9QFI5	Diazepam-binding inhibitor	0.5201	0.3399	0.6268	0.5566	down	P07108	Acyl-CoA-binding protein
P52722	Metallothionein-1	0.2638	0.3343	0.6783	0.5523	down	_	
B8JI17	Prosaposin	1.4011	0.5255	0.5526	0.4840	down	P07602	Prosaposin
A8DZ70	Metadherin a	0.5932	0.5322	0.6659	0.8409	down	Q86UE4	Protein LYRIC
Q5RI57	Novel protein	1.2189	0.6343	0.4838	0.4314	down	P14222	Perforin-1
Q7SXM1	Cytochrome c oxidase subunit	0.5713	0.6517	0.4349	1.0476	down	P14854	Cytochrome c oxidase subunit 6B1
A3KPG5	Pentaxin	1.5395	1.6500	1.5888	1.6188	up	P02741	C-reactive protein
Q7ZUM0	Tyrosine 3-monooxygenase/tryptophan 5-monooxygenase activation protein, theta polypeptide a	1.2742	1.5503	1.5686	1.7338	up	P63104	14-3-3 protein zeta/delta
Q6P3G9	Endoplasmic reticulum protein 44	1.5701	1.5026	1.2002	1.8709	up	Q9BS26	Endoplasmic reticulum resident protein 44
F1R7E6	Iodothyronine deiodinase	0.4536	1.6392	1.7741	1.9650	up	P49895	Type I iodothyronine deiodinase
Q6PBJ8	Peptidylprolyl isomerase	0.6250	1.5122	2.4524	2.0269	up	P68106	Peptidyl-prolyl cis-trans isomerase FKBP1B
F1R5X7	Dehydrogenase/reductase (SDR family) member 11b	1.2716	1.6556	1.8453	2.1344	up	Q6UWP2	Dehydrogenase/reductase SDR family member 11
F1QWR0	Nipsnap homolog 3A (C. elegans)	1.1399	1.9640	1.8741	2.4264	up	Q9UFN0	Protein NipSnap homolog 3A
Q6NYL5	Alanine aminotransferase 2-like	1.1524	1.6657	2.0939	2.6848	up	Q8TD30	Alanine aminotransferase 2

A5PN40	Crystallin, zeta (quinone reductase)	0.8716	1.7176	2.2955	2.7897	up	Q08257	Quinone oxidoreductase
E7F1Y3	Si:zfos-411a11.2	0.8300	1.6838	1.8907	2.9921	up	Q16696	Cytochrome P450 2A13
A0A0R4IPA1	Calcium-activated nucleotidase 1b	1.1865	2.4563	1.9018	3.1827	up	Q8WVQ1	Soluble calcium-activated nucleotidase 1
A0A0R4INN3	Alanine--glyoxylate aminotransferase 2	0.9062	1.9414	2.7136	3.5599	up	Q9BYV1	Alanine--glyoxylate aminotransferase 2
Q6DRD2	26S protease regulatory subunit S10B	0.6337	1.5174	3.1536	3.8520	up	P62333	26S proteasome regulatory subunit 10B
F1QCE3	Peroxiredoxin 5	0.7044	2.9685	3.1566	4.2799	up	P30044	Peroxiredoxin-5, mitochondrial
Q90485	Hemoglobin subunit beta-2	8.0481	1.2324	1.6037	9.4099	up	P02100	Hemoglobin subunit epsilon
F8W5T8	Zgc:152945	0.4632	3.5085	3.3849	4.7427	up	P02790	Hemopexin

Figure S1. Verification of protein pattern duplication efficiency. (a) Ratio correlation between the duplicated experiments, person correlation (r) visualized as scatterplot. (b) Protein profiling by SDS-PAGE.

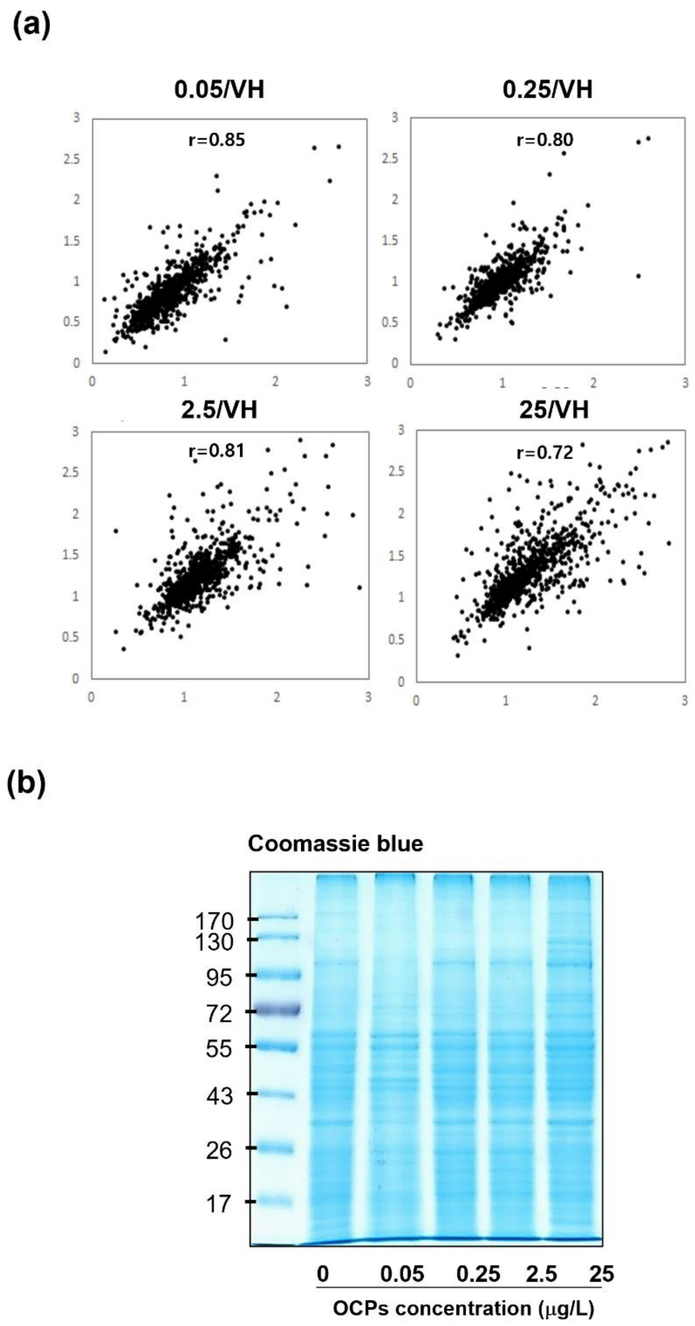

Figure S2. (A) Gene ontology (GO) enrichment analysis and (B) KEGG pathway- and Interpro-based enrichment analysis for proteins consistently up- and down-regulated at all tested concentrations.

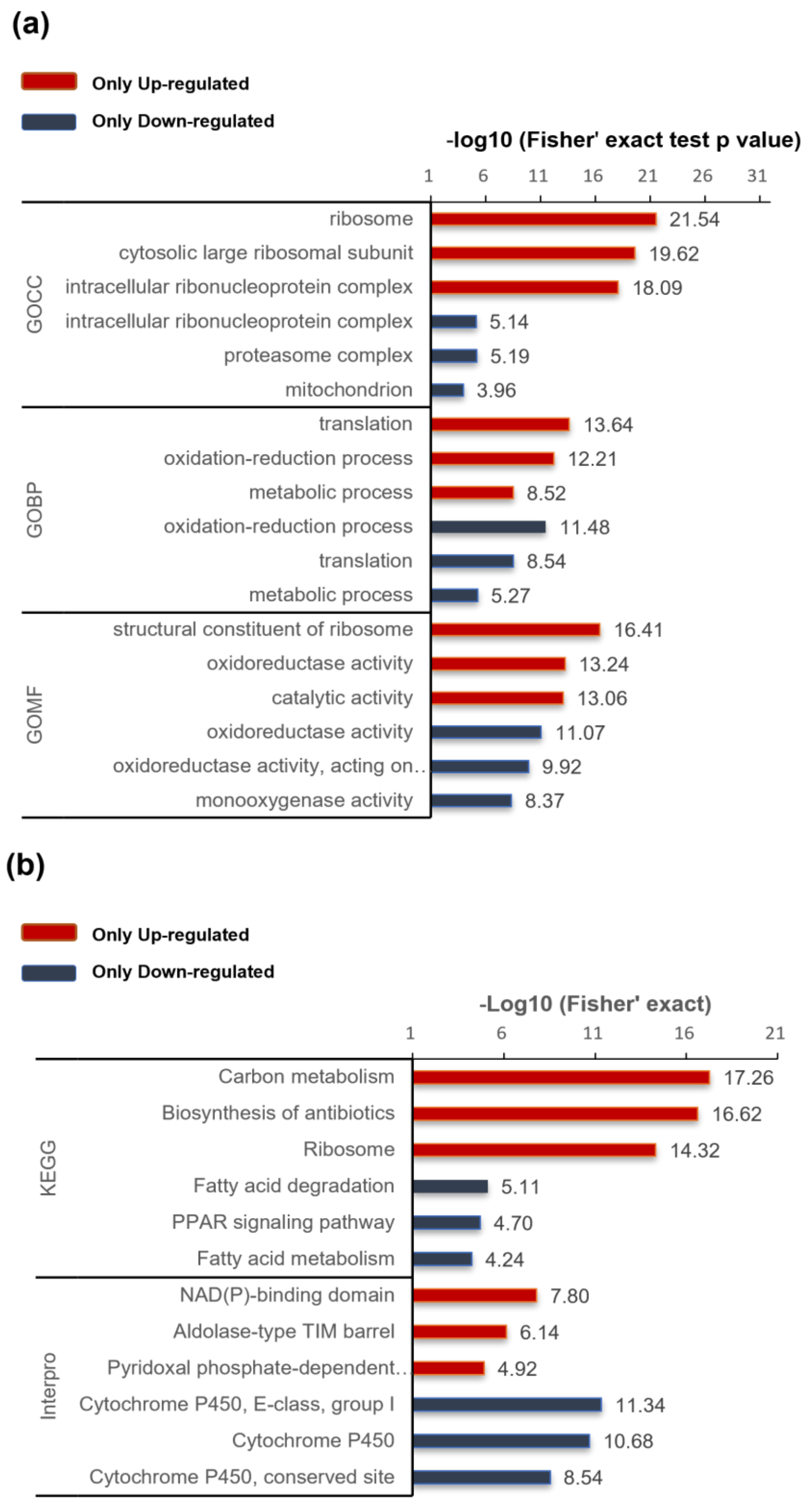

Figure S3. GO enrichment analysis of proteins in cluster 3. The Fisher's exact test P value was used to evaluate the enrichment efficiency.

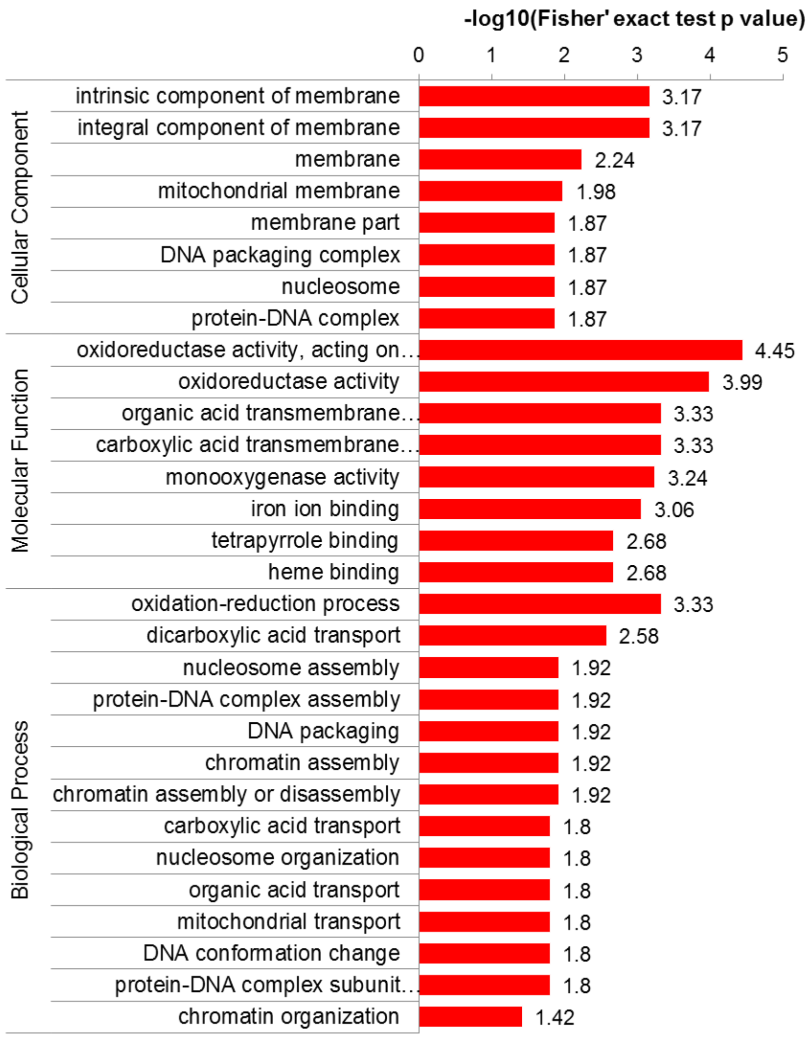

Figure S4. The functional protein association networks of differentially expressed proteins (a) up-regulated and (b) down-regulated at 0.05 μg/L compared to control. This network was mapped in the STRING v.11.0 with a high confidence value of 0.9. The proteins and its connectivity are described by nodes and lines, respectively.

(a) Up-regulated (0 vs 0.05)

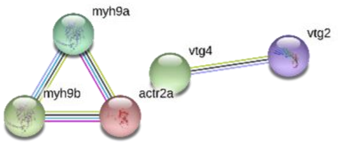

(b) Down-regulated (0 vs 0.05)

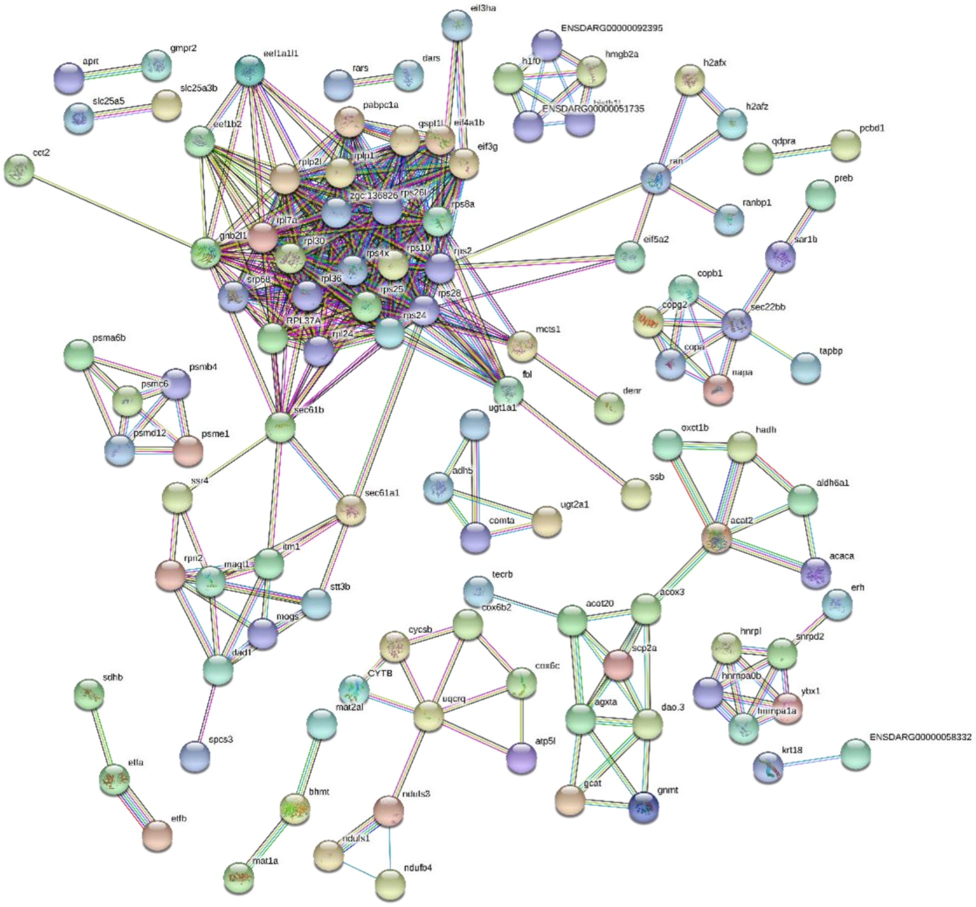

Supplement: Supplementary file 1 [file ijerph-19-04991-s001.zip › ijerph-1642717-SI.pdf]
